# Differentially Expressed Gene Transcripts Using RNA Sequencing from the Blood of Immunosuppressed Kidney Allograft Recipients

**DOI:** 10.1371/journal.pone.0125045

**Published:** 2015-05-06

**Authors:** Casey Dorr, Baolin Wu, Weihua Guan, Amutha Muthusamy, Kinjal Sanghavi, David P. Schladt, Jonathan S. Maltzman, Steven E. Scherer, Marcia J. Brott, Arthur J. Matas, Pamala A. Jacobson, William S. Oetting, Ajay K. Israni

**Affiliations:** 1 Minneapolis Medical Research Foundation, Minneapolis, Minnesota, United States of America; 2 Department of Medicine, Hennepin County Medical Center, University of Minnesota, Minneapolis, Minnesota, United States of America; 3 Division of Biostatistics, University of Minnesota, Minneapolis, Minnesota, United States of America; 4 College of Pharmacy, University of Minnesota, Minneapolis, Minnesota, United States of America; 5 Department of Medicine, Perelman School of Medicine, University of Pennsylvania, Philadelphia, PA, United States of America; 6 Department of Molecular and Human Genetics, Baylor College of Medicine, Dallas, Texas, United States of America; 7 Department of Surgery, University of Minnesota, Minneapolis, Minnesota, United States of America; 8 Department of Epidemiology and Community Health, University of Minnesota School of Medicine, Minneapolis, Minnesota, United States of America; UNIFESP Federal University of São Paulo, BRAZIL

## Abstract

We performed RNA sequencing (RNAseq) on peripheral blood mononuclear cells (PBMCs) to identify differentially expressed gene transcripts (DEGs) after kidney transplantation and after the start of immunosuppressive drugs. RNAseq is superior to microarray to determine DEGs because it’s not limited to available probes, has increased sensitivity, and detects alternative and previously unknown transcripts. DEGs were determined in 32 adult kidney recipients, without clinical acute rejection (AR), treated with antibody induction, calcineurin inhibitor, mycophenolate, with and without steroids. Blood was obtained pre-transplant (baseline), week 1, months 3 and 6 post-transplant. PBMCs were isolated, RNA extracted and gene expression measured using RNAseq. Principal components (PCs) were computed using a surrogate variable approach. DEGs post-transplant were identified by controlling false discovery rate (FDR) at < 0.01 with at least a 2 fold change in expression from pre-transplant. The top 5 DEGs with higher levels of transcripts in blood at week 1 were *TOMM40L*, *TMEM205*, *OLFM4*, *MMP8*, and *OSBPL9* compared to baseline. The top 5 DEGs with lower levels at week 1 post-transplant were *IL7R*, *KLRC3*, *CD3E*, *CD3D*, and *KLRC2* (Striking Image) compared to baseline. The top pathways from genes with lower levels at 1 week post-transplant compared to baseline, were T cell receptor signaling and iCOS-iCOSL signaling while the top pathways from genes with higher levels than baseline were axonal guidance signaling and LXR/RXR activation. Gene expression signatures at month 3 were similar to week 1. DEGs at 6 months post-transplant create a different gene signature than week 1 or month 3 post-transplant. RNAseq analysis identified more DEGs with lower than higher levels in blood compared to baseline at week 1 and month 3. The number of DEGs decreased with time post-transplant. Further investigations to determine the specific lymphocyte(s) responsible for differential gene expression may be important in selecting and personalizing immune suppressant drugs and may lead to targeted therapies.

## Introduction

Kidney allograft transplantation is the most cost-effective treatment for end stage renal disease [1,2,3]. Unfortunately, the long-term success of transplantation is often threatened by acute rejection (AR) and chronic allograft dysfunction (CGD), which are common adverse outcomes in kidney allograft recipients despite modern immunosuppression [4]. Acute rejection occurs early post-transplant and may be antibody [5] or T-cell mediated [6]. Chronic allograft dysfunction is irreversible [4] with no effective treatments [7,8]. Thus, highly effective prophylactic immunosuppressive therapy is critical in preventing AR and CGD. Despite the use of better immunosuppressive regimens today than 15 years ago, lymphocytes, the primary targets of immunosuppressive drugs, still find ways to evade the immune suppression. This may be due to altered genetic mechanisms and cellular pathways that lead to insufficient T and/or B-cell suppression. To address if genetic mechanisms may be related to drug related immunosuppression we investigated if gene expression changes occur before and after the start of immune suppressant therapy and over time as therapy changes. We believe that eventually gene signatures can be used to personally tailor immune suppression therapies and predict clinical outcomes.

This study is the first to describe DEGs over time using whole transcriptome sequencing of PBMCs from kidney allograft recipients who have not developed AR within the first 7 months post-transplant. Previous microarray studies have focused on individuals with rejection events and have identified genes associated with AR by analyzing RNA isolated from donor kidney allograft biopsies [9,10,11]. PBMCs have also been used to identify DEGs in kidney transplant recipients using gene sets [12], or microarrays [11,12,13,14] to identify AR signatures. These previous studies have allowed for a better understanding of the biology of transplant rejection. However, RNAseq is a new and superior method to identify DEGs and associated molecular cellular pathways since it is not limited to available probes, has increased sensitivity [15], and detects alternative splice variants, can detect low level expression [16] and previously unknown transcripts.

Most studies using microarrays showed gene expression changes at the time of a rejection event. However, other factors, such as the immunosuppression drug regimen, are also likely associated with changes in transcript expression. Most transplant centers reduce immunosuppression at 2 to 3 months post-transplant and it is likely that these changes in maintenance immune suppression alter expression. We report here gene expression changes in the blood of patients without AR or CGD; therefore the observed DEGs are not those associated with clinically evident rejection events. This analysis is the first step identifying gene signatures that correlate with favorable immune suppression. The ultimate goal is to identify an optimal immune genetic signature and ultimately personalize immunosuppressant drugs regimens to achieve that signature. *Our hypothesis is that PBMC transcripts vary after the initiation of immunosuppression and at different times following kidney allograft transplantation*. To identify these DEGs, we performed RNAseq analysis on PBMCs to identify gene expression patterns prior to transplant, 1 week, 3 months, and 6 months post-transplant in kidney allograft recipients. We identified DEGs that will further our understanding of the physiological, cellular and molecular mechanisms of favorable immune suppression and kidney transplantation. Ideally, these expression patterns will lead to extending allograft survival and in turn improve the quality and longevity of kidney recipient lives.

## Methods

### Patients

Thirty-two adults receiving living donor kidney allografts were studied. Patients received thymoglobulin induction and maintenance therapy with tacrolimus or cyclosporine, with mycophenolate and short course steroids to days 5–7 post-transplant. Four of the patients received tacrolimus or cyclosporine prior to transplantation. Five patients were receiving steroids and 9 were receiving mycophenolate at baseline for underlying disease. The subjects had no rejection or any previous rejection at time of each sample collection. Sequential whole blood samples for isolation of PBMCs were collected at baseline (pre-transplant, n = 32), week 1 (n = 31), month 3 (n = 18) and month 6 (n = 15) post-transplant. Some samples were not obtained because patients did not return to the transplant clinic for follow-up center visits and clinical follow ups were performed by referring physician. All patients in this study provided written informed consent following protocol that was approved by the institutional review board of the University of Minnesota.

### RNA Sequencing

Blood was collected into BD Vacutainer EDTA coated tubes. RNA was isolated from approximately 12 mLs of whole blood PBMCs using the Qiagen QIAamp RNA Blood Mini kit (Germantown, MD) within 2 hours of blood draw. RNA was quantitated with a Nanodrop 800 spectrophotometer. One μg of each RNA sample was used to prepare RNAseq libraries based on the method as outlined by Zhong and colleagues [17] with modifications and added quality checks. Sample quantity and integrity was checked using RiboGreen analysis and an Agilent 2100 Bioanalyzer or Caliper equivalent. Each sample passing quality control (RNA mass > 1 μg and RNA Integrity Number > 6) was used to create a polyA^+^ stranded barcoded RNAseq library using standard protocols. Five samples were pooled for each lane for Illumina Hi-seq 2000 sequencing to generate 20–40 million mapped paired-end reads per sample.

### Data Analysis

Quality control of fastq data was performed using FastQC:Read QC [18]. The 170 bp paired-end reads were then aligned to human genome (GRCh37/hg19 assembly) via Tophat2 using the iGenome human UCSC reference annotation [19,20,21] to align and quantitate the transcripts. Transcript assembly and transcript abundance was determined using Cufflinks program [20] where the hg19_genes_2012-03-09.gtf file was used as reference annotation guide to determine fragments per kilobase per million reads (FPKM) for each transcript. The raw FPKM values were normalized following a previously described procedure [22]. Principal components (PCs) were computed using the surrogate variable approach [23]. We analyzed the log transformed normalized FPKM values using a linear mixed effects model adjusting for age, gender and the first two PCs. We tested for gene effect using the Kenward-Roger approximate F-test to account for the potential impact of small sample size [24]. DEGs were identified by controlling false discovery rate (FDR) at <0.01 accounting for 15,669 genes. These genes were in the top 75% of variation of expression and this was done to remove genes with low levels of variation. DEGs were those with a 2 or greater fold change (up or down) in expression, compared to pre-transplant expression. DEGs were further investigated for function and pathway enrichment using Ingenuity Pathway Analysis by analyzing genes with higher levels than baseline, lower levels, or combined higher and lower level genes at each time point.

## Results

### Transcripts expression over time after transplant

Patient characteristics are described in Table 1.

**Table 1:** Kidney Recipient and Donor Characteristics.

Characteristic	Frequency, n	Percent
**Recipients**		
Age years (mean +/- Standard Deviation)	48.2 +/- 14.4 years	
Gender		
Female	9	28.1
Male	23	71.9
Primary Disease		
Diabetes	7	21.9
Glomerular Disease	8	25.0
Hypertension	3	9.3
Polycystic Disease	6	18.8
Other	8	25.0
Race		
Native American or Alaskan Decent	3	9.4
African Decent	2	6.2
European Decent	27	84.4
Transplant Type		
Kidney	31	96.9
Simultaneous Pancreas and Kidney	1	3.1
Immunosuppression at week 1		
Tacrolimus	21	65.6
Cyclosporine	11	34.4
Mycophenolate	31	96.9
Steroids at week 1		
Yes	6	18.8
No	26	81.2
Steroids at baseline		
Yes	5	15.6
No	27	84.4
Immunosuppression at Baseline		
Tacrolimus	2	6.3
Cyclosporine	2	6.3
Mycophenolate	9	28.1
**Donors**		
Age years (mean +/- Standard Deviation)	41.1+/- 11.7 years	
Gender		
Female	18	56.2
Male	14	43.8
Type		
Living Unrelated Donor	14	43.8
Living Relative Donor	18	56.2
Race		
Native American or Alaskan Decent	1	3.1
African Decent	3	9.4
European Decent	28	87.5
CMV status		
Negative Recipient, Negative Donor	9	28.1
Negative Recipient, Positive Donor	5	15.6
Positive Recipient	18	56.3

### Alignment and mapping of RNA sequencing reads

Fastq files of all 96 samples passed FastQC report with good per base sequence quality, per base sequence quality score, per base N content and good sequence length distribution. RNA sequencing paired-end reads of approximately 170 bp were aligned to the reference human genome using the TopHat2 algorithm with results summarized in Table 2. In total, there were 96 paired-end reads aligned with an average overall alignment rate of 89.9%. There were 23,177 genes with FPKM greater than zero and of these 7,567 genes had an average FPKM greater than 5.

**Table 2:** Alignment of paired end RNAseq reads with Reference Genome, n = 96.

	Overall Alignment	Aligned pairs
**Average**	89.9%	31,496,089
**Standard deviation**	0.02	7,691,470
**Median**	90.2%	30,123,106

RNA sequencing reads were aligned to human genome (GRCh37/hg19 assembly) via Tophat2.

Compared to pre-transplant, the number of DEGs declined over time as 500 DEGs were present at week 1, 268 at month 3 and 87 at month 6 (Table 3). Additionally, at month 3 post-transplant there were more genes with lower levels than higher levels compred to baseline (Table 3). At month 6, there were more DEGs with higher levels than DEGs with lower levels compared to baseline (Table 3).

**Table 3:** Differentially expressed genes (DEG) after transplant compared to baseline at pre-transplant.

Gene Expression	Post-Transplant DEGs compared to baseline (FDR< 0.01)		
	1 Week (n = 31)	3 Months (n = 18)	6 Months (n = 15)
**No. genes with higher levels** (2 or greater fold change)	191	94	50
**No. genes with lower levels** (2 or greater fold change)	309	174	37
**All genes with higher and lower levels** (2 or greater fold change)	500	268	87

n = number of subjects per time point.

**Fig 1:**
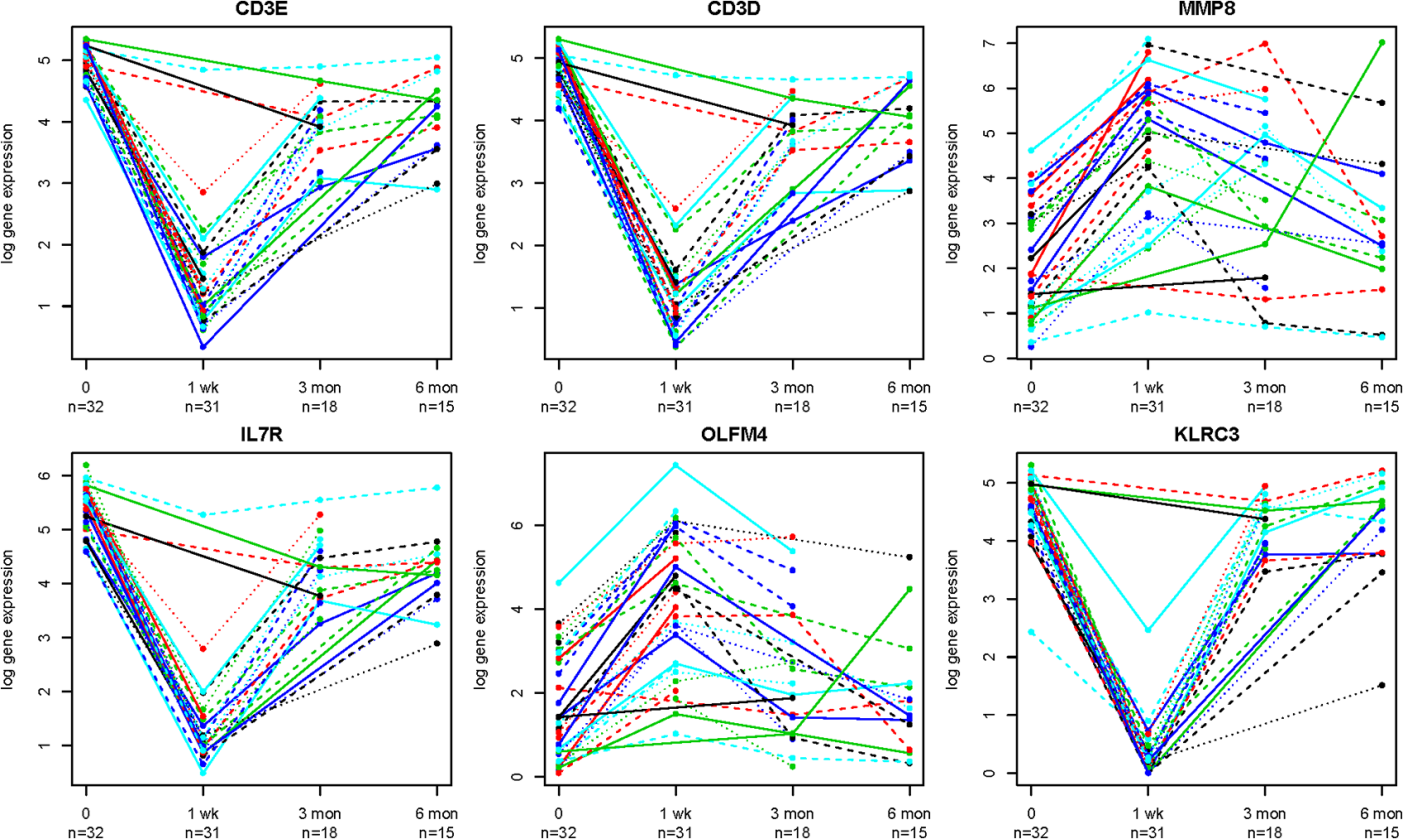
Expression of representative genes over time in kidney transplant recipients. Representative time series of fold expression changes relative to baseline (time 0) of some top genes with higher and lower level compared to baseline. Each line on each graph represents the expression of the particular gene in a separate kidney allograft recipient. Note that all patients do not have data all the time points. CD3E = CD3 Epsilon TCR complex; CD3D = CD3 Delta TCR complex; MMP8 = Matrix Metallopeptidase 8; IL7R = Interleukin 7 Receptor; OLFM4 = Olfactomedin 4; KLRC3 = Killer Cell Lectin-like Receptor subfamily C, member 3.

Table 4 lists the top DEGs at each time post-transplant and Fig 1 shows selected genes with expression over time. Tables with genes names, FDR, p-values and fold changes at each time post-transplant are in supplementary data (S1–S6 Tables). Some of the genes with lower levels than baseline, with the smallest FDR values and highest fold change in levels were not in well annotated genes because RNAseq identified previously unknown transcripts such as microRNA 650 and open reading frames such as C1orf204 or C10orf105. However, the majority of the identified DEGs had known functions. Additionally, the majority of micro RNAs were not captured due to polyA stranded RNAseq library construction.

**Table 4:** Top Differentially Expressed Genes Post-transplant Following Kidney Transplant compared to baseline with FDR < 0.01 and 2 or greater fold change.

Week 1			Month 3			Month 6		
Gene ID	Entrez gene name	Fold change	Gene ID	Entrez gene name	Fold change	Gene ID	Entrez gene name	Fold change
**Higher Levels Than at Baseline**								
**TOMM40L**	translocase of outer mitochondrial membrane 40 homolog (yeast)-like	18.20	**TOMM40L**	translocase of outer mitochondrial membrane 40 homolog (yeast)-like	10.64	**OSBPL9**	oxysterol binding protein-like 9	4.63
**TMEM205**	transmembrane protein 205	14.37	**TMEM205**	transmembrane protein 205	6.48	**CD300LD**	CD300 molecule-like family member d	4.22
**OLFM4**	olfactomedin 4	10.73	**OSBPL9**	oxysterol binding protein-like 9	6.38	**HBB**	hemoglobin, beta	4.09
**MMP8**	matrix metallopeptidase 8 (neutrophil collagenase)	9.24	**UBXN11**	UBX domain protein 11	5.36	**NREP**	neuronal regeneration related protein	3.91
**OSBPL9**	oxysterol binding protein-like 9	8.55	**MMP8**	matrix metallopeptidase 8	4.69	**HBA2**	hemoglobin, alpha 1	3.90
**LTF**	Lactotransferrin	7.93	**NREP**	neuronal regeneration related protein	4.60	**BMP6**	bone morphogenetic protein 6	3.82
**AZU1**	azurocidin 1	7.88	**CD177**	CD177 molecule	4.20	**HBA1**	hemoglobin, alpha 1	3.82
**CRISP3**	cysteine-rich secretory protein 1	7.85	**OLFM4**	olfactomedin 4	3.95	**MNF1**	Ubiquinol-cytochorome C reductase complex assembly factor 2	3.71
**BPI**	bactericidal/permeability-increasing protein	7.73	**HP**	haptoglobin	3.87	**TBC1D8**	TBC1 domain family, member 8 (with GRAM domain)	3.20
**UBXN11**	UBX domain protein 11	7.58	**CRISP3**	cysteine-rich secretory protein 1	3.80	**SYVN1**	synovial apoptosis inhibitor 1, synoviolin	3.15
**Lower Levels than at Baseline**								
**IL7R**	interleukin 7 receptor	-17.73	**C1orf204**	chromosome 1 open reading frame 204	-14.13	**ELK2AP**	ELK2A, member of ETS oncogene family, pseudogene	-12.25
**KLRC3**	killer cell lectin-like receptor subfamily C, member 3	-16.61	**VSIG8**	V-set and immunoglobulin domain containing 8	-14.13	**PIK3IP1**	phosphoinositide-3-kinase interacting protein 1	-11.41
**CD3E**	CD3e molecule, epsilon (CD3-TCR complex)	-14.99	**PIK3IP1**	phosphoinositide-3-kinase interacting protein 1	-7.67	**C1orf204**	chromosome 1 open reading frame 204	-10.21
**CD3D**	CD3d molecule, delta (CD3-TCR complex)	-14.39	**C10orf105**	chromosome 10 open reading frame 105	-5.37	**VSIG8**	V-set and immunoglobulin domain containing 8	-10.21
**KLRC2**	killer cell lectin-like receptor subfamily C, member 2	-14.27	**IL7R**	interleukin 7 receptor	-5.29	**STAG3**	stromal antigen 3	-6.49
**KLRB1**	killer cell lectin-like receptor subfamily B, member 1	-13.78	**LEF1**	lymphoid enhancer-binding factor 1	-5.16	**MIR650**	microRNA 650	-5.38
**KLRC1**	killer cell lectin-like receptor subfamily C, member 1	-13.51	**MAL**	mal, T-cell differentiation protein	-4.94	**C10orf105**	chromosome 10 open reading frame 105	-4.93
**CD3G**	CD3g molecule, gamma (CD3-TCR complex)	-11.94	**CD3E**	CD3e molecule, epsilon (CD3-TCR complex)	-4.65	**EPHB1**	EPH receptor B1	-4.87
**LEF1**	lymphoid enhancer-binding factor 1	-11.71	**TCF7**	transcription factor 7 (T-cell specific, HMG-box)	-4.60	**IGLL5**	immunoglobulin lambda-like polypeptide 1	-4.81
**ITK**	IL2-inducible T-cell kinase	-10.82	**CD3D**	CD3d molecule, delta (CD3-TCR complex)	-4.60	**IL8**	chemokine,ligand 8	-4.31

### Functional annotation of DEGs and Ingenuity Pathway Analysis

#### One week post-transplant

Functional annotation of DEGs with higher and lower levels with FDR < 0.01 and 2 or greater fold change compared to baseline, indicated that the top transcripts were in T-cell related pathways (Table 5). This may be related to patients receiving T-cell depletion induction with thymoglobulin and a calcineurin inhibitor in the immediate peritransplant period. Specifically, the top altered pathway was T-cell receptor signaling (Table 5) based on 18 genes with lower levels than at baseline (S7A Table). This pathway primarily contained the CD3D, CD3E, and CD3G genes for T-cell receptor signaling components. When all significantly higher and lower level DEGs compared to baseline were analyzed together no pathways based on higher level genes were identified due to a masking of the effect by the greater number of lower level genes (309 DEGs) compared to higher level genes (191 DEGs). Thus, we investigated only the DEGs that had increased levels in blood compared to baseline, separately with pathway analysis and identified the axonal guidance signaling pathway containing 12 genes (Table 5 and S7A Table). Interestingly, matrix metalloprotease (MMP) genes, were involved in all three of the top identified pathways at week 1 (S7A Table and S8 Table), indicating that the MMP9 (S7A Table and S8 Table) and MMP8 (Table 4) are likely important in kidney transplants without rejection.

**Table 5:** Top altered pathways from blood following kidney transplant.

Week 1	Month 3	Month 6
**Higher Levels Than at Baseline**		
Axonal Guidance Signaling (12)	IL-8 Signaling (6)	Antiproliferative Role of Somatostatin Receptor 2 (2)
LXR/RXR Activation (6)	Complement System (3)	Spermine and Spermidine Degradation I (1)
Airway Pathology in Chronic Obstructive Pulmonary Disease (2)	Airway Pathology in Chronic Obstructive Pulmonary Disease (2)	Melatonin Degradation II (1)
**Lower Levels Than at Baseline**		
T Cell Receptor Signaling (18)	T Cell Receptor Signaling (16)	Granulocyte Adhesion and Diapedesis (8)
iCOS-iCOSL Signaling in T Helper Cells (17)	iCOS-iCOSL Signaling in T Helper Cells (16)	Agranulocyte Adhesion and Diapedesis (8)
CTLA4 Signaling in Cytotoxic T Lymphocytes (13)	CTLA4 Signaling in Cytotoxic T Lymphocytes (12)	Communication between Innate and Adaptive Immune Cells (6)
**Higher and Lower Levels, Combined**		
T Cell Receptor Signaling (18)	T Cell Receptor Signaling (16)	Granulocyte Adhesion and Diapedesis (17)
iCOS-iCOSL Signaling in T Helper Cells (17)	iCOS-iCOSL Signaling in T Helper Cells (16)	Agranulocyte Adhesion and Diapedesis (14)
CTLA4 Signaling in Cytotoxic T Lymphocytes (14)	CTLA4 Signaling in Cytotoxic T Lymphocytes (12)	Macropinocytosis Signaling (8)

Top canonical gene pathways altered at week 1, months 3 and 6 compared to baseline using Ingenuity Pathway Analysis of DEGs with false discovery rate (FDR) < 0.01 and 2 or greater fold change. The number of DEGs in the pathway is shown in parentheses.

DEG = Differentially Expressed Gene.

Baseline = prior to transplant.

#### Three months post-transplant

Compared to 1 week post-transplant, similar genes were altered at month 3 (Table 4). However, the number of DEGs at month 3 was substantially lower than at 1 week with 94 genes with increased levels compared to baseline and 174 genes with decreased levels (Table 3). T-cell receptor signaling remained the top pathway altered with 16 involved genes (Table 5). However, the fold changes in gene expression were much lower than at week 1 (Table 4). For instance, the CD3D gene was -14.39 fold change at week 1 compared to -4.65 fold change at month 3 (Table 4). This suggests that the expression of some of the genes in the early affected pathways may be gradually returning to pre-transplant expression levels. The second pathway with lower levels than baseline was iCOS-iCOSL signaling in T-helper cells with 16 genes involved. The upregulated pathways at month 3 were slightly different than compared to week 1. The expression pattern indicates that the complement system was activated as well as IL-8 signaling. The genes MMP8 and MMP9 remained at higher levels at month 3 compared to baseline. Interestingly, pathway analysis also identified both the granulocyte adhesion and diapedesis and the agranulocyte adhesion and diapedesis pathways at month 3 among genes with higher levels compared to baseline (S4 Table). This suggests that perhaps by month 3, leukocytes were leaving the blood vessels and potentially extravasating into kidney allograft as part of the immune response.

#### Six months post-transplant

By 6 months post-transplant, the numbers of DEGs compared to baseline were severely reduced compared to week 1 or month 3 post-transplant. At month 6, only 87 genes were differentially expressed compared to baseline with 50 at higher levels and 37 at lower levels. Remarkably, at month 6, the top pathways have changed and now granulocyte adhesion and diapedesis and the agranulocyte adhesion and diapedesis pathways were among genes with lower levels compared to baseline (Table 5). This pathway was primarily composed of chemokines genes (S7C Table and S8 Table).

Gene expression changes over time in representative genes are shown in Fig 1. In most cases, genes at higher levels than baseline remained that way or were moving back to baseline transcript levels. However, some genes showed different patterns of expression over time. For example the granulocyte and agranulocyte adhesion, and diapedesis pathways were at higher levels at month 3 (S8 Table) and then at lower levels at month 6 (S7C Table and S8 Table). This was unusual in our study where a pathway was at higher levels after transplant compared to baseline and then at lower levels at the next time point. Thus, the data indicate gene expression and pathways can change as a function of time following transplant and this is visualized in Fig 1. At month 6, we also observed lower levels of genes involved in T-cell signaling pathways which were possibly due to the standard reduction in immune suppressant drugs that occurs after month 3 and a gradual T-cell recovery which occurs as induction therapy effects wane.

## Discussion

This is the first study to conduct whole transcriptome sequencing in PBMCs and characterize changes in expression at multiple times post-transplant. This research design is distinctly unique compared to other studies using microarray of kidney biopsies or blood at one time point following transplant usually at the time of a rejection event. As we hypothesized, PBMC transcripts vary after the initiation of immunosuppression and at different times following kidney allograft transplantation. We showed that many genes had altered expression levels at week 1 and then slowly move towards baseline expression levels as time passes post-transplant (Fig 1). Of major importance, our data show there are substantial transcript expression changes in the blood of patients not experiencing rejection events. For instance, the T-cell signaling components CD3D, CD3E, and CD3G had decreased levels at week 1 post-transplant (Table 4), but levels increase towards pre-transplant levels at months 3 and 6 in the blood (Fig 1). Speculatively, thymoglobulin induction therapy may be depleting T-cells in the blood that typically express CD3E, CD3G and CD3D. Most pathways among genes with lower levels compared to baseline were T-cell related possibly due to T-cell depleting induction agents or calcineurin inhibitor therapy early after transplant, but the up regulated pathways involve genes in axonal guidance or complement activation. It is possible that axonal guidance genes are involved in signaling the leukocytes or as a cross talk mechanism with the nervous system. However, since our patients did not have rejection, this pathway may represent an allograft tolerance mechanism. Many genes with lower levels compared to baseline, such as killer cell lectin receptors or CD3 signaling components at week 1 (Table 4), or genes with higher levels, such as MMP8 at week 1 or chemokines at month 6 (Table 4) could be expressed by specific cell types. Therefore, it is possible that the expression we observed are due to changes in the abundance of certain lymphocyte subtypes in the blood which express these transcripts. It is important that in the future we understand which lymphocytes are responsible for the altered transcripts and their relative frequency in the blood.

It is difficult to directly compare our results to published gene expression data since ours is the first study using RNAseq longitudinally in non-rejecting kidney patients’ blood. Many patients at baseline were not on immunosuppression (Table 1). Comparison of gene expression among the baseline patients on immunosuppression (n = 13) versus those not on immunosuppression (n = 19) did not show any significantly different genes at FDR < 0.01 (data not shown). One previous study did investigate, longitudinally (years 1, 2, 3, and 4 post-transplant) RNA expression by microarray in biopsy and blood [25], but it is not directly comparable as the study used experimental donor hematopoietic stem cells to induce tolerance in HLA-identical transplants. Another study by Sarwaal and colleagues used microarray and qRT-PCR to detect a set of genes for AR called a kSORT assay [5,12,26]. Not surprising, significant genes in our study were not the same as those identified in their analysis. However, they identified the gene IL7R to be associated with AR [26,27] which we identified as one of the genes with lower levelsat week 1 and then increased levels at month 3 post-transplant, compared to baseline. IL7R is a cytokine receptor expressed by naïve and memory T-cells. This is consistent with down regulation in T-cell signaling pathways due to immune suppression induced depletion of T-cells. Another study conducted on leukocytes and biopsies of kidney transplant recipients identified some of the same genes we identified (CD3D, CCL5, LTF, LCN) but they used a microarray platform [11]. CD3D was found to have increased levels in the biopsies of AR patients [11] possibly indicating cells that express CD3D invade the kidney during an AR event. Additionally, Halloran and colleagues conducted microarray studies on kidney biopsies to determine which cells are present in the allograft at the time of AR. Five transcripts identified in the kidney biopsies were T-cell specific including CD3D, TCRA, CXCR6, GPR171 and NELL2 [28]. Specifically, the T-cell related transcripts were present in kidney biopsies with T-cell mediated rejection. Thus, both studies [11,28] indicate CD3D expressing cells are present in the kidney during an AR event. However, in our study, CD3D levels were lower in the blood (Fig 1, Table 4) of our transplant recipients without rejection, which may mean the CD3D expression, is lower in blood during optimum immune suppression. Thus, CD3D is likely an important biomarker of immunosuppression in kidney allograft patients.

Like us, Halloran also identified LTF and OLFM4 to have increased levels after transplant, but they found these genes in biopsy tissue, and associated them with injury repair response transcripts [29]. They also identified LCK, a T-cell restricted kinase [30], MMP9 and RNASE3 [31]. Interestingly, MMP9 is associated with the previously undescribed axonal guidance signaling pathway that we identified at increased levels following transplant, compared to baseline. Genes involved in the axonal guidance signaling pathway at week 1 post-transplant include ABLIM3, ADAM15, BMP6, GNG11, MMP8, MMP9, MYL9, PLXNB2, PRKAR2B, TUBB1, TUBB6, WNT5B (S7A Table). The axonal guidance signaling pathway suggests that even in the presence of immunosuppression, the immune cells were still attempting to migrate into the allograft and possibly investigate the new allograft antigens. Calcineurin based immunosuppression targets T cell receptor signaling and may have less effect on axonal guidance signaling pathways. Thus, the sensitivity and time series analysis using RNAseq has led us to the identification of pathways and genetic signatures previously not reported.

Our study has several limitations. This study was conducted in a single center, while a multicenter approach would be more generalizable. Additionally, we only have non-rejecting kidney recipients in our study up to 7 months post-transplant, and it is possible some of the patients had AR or CGD at a later time point when their immunosuppression is lowered. Future studies should compare our findings in patients without rejection to those with kidney AR or CGD. Nonetheless, our study does establish a possible gene expression profile in kidney transplant patients with apparent optimal immune suppression. Some patients did not have gene expression at all the 3 time points of baseline, 1 week, and 3 months post-transplant. However, for the top genes, the gene expression profile for patients with all three time points did not appear different (S1 Fig). An additional limitation of this study is that we used polyA stranded RNAseq libraries which do not account for most microRNAs and other non-polyA transcripts. Also, we use a standard annotated genome that does not specifically account for alternative splice variants. We also did not validate our results with qRT-PCR due to the limited sample of RNA from the patients. Furthermore, our study uses PBMCs isolated from whole blood to determine DEGs following kidney transplant. However we did not determine the cell types present in PBMCs and distinguish what cell types in the PBMCs were responsible for the changing abundance of transcripts in the blood. It is possible that the cells that express the particular transcripts are in lower abundance in the peripheral blood leading to lower levels of the cell-specific RNAs. In contrast, RNAs that appear at higher levels in the PBMCs could indicate that cells, that expressed those specific RNAs, are proliferating leading to increased cell-specific transcript abundance. It is thus important in the future to determine what cells are most responsible for alterations in transcript abundance in the blood so that we can identify cell type specific gene expression signatures and molecular cellular mechanisms of kidney allograft transplantation. Lastly, we did not account for all baseline clinical factors in a multivariate model due to the small sample size, some of which could confound our findings. Alternatively, we used a surrogate variable approach to approximate potential confounders.

We report the first use of RNAseq, to detect the transcriptional changes in PBMCs at multiple time points following kidney transplantation. RNAseq is superior to microarray to determine DEGs because it’s not limited to available probes and has increased sensitivity to detect low level transcript expression [16]. This is important because the majority of the transcripts early post-transplant with at least a 2 fold change in expression were in lower levels compared to baseline. Also, RNAseq can identify transcripts that may not have a probe on a microarray chip. Therefore, RNAseq is a more precise method of characterizing gene expression in transplantation than microarray. This study leads to better understanding of the molecular genetic and cellular pathways that are associated with kidney transplantation without clinical rejection. We also show that genetic signatures change as a function of time following transplant. This study also establishes the feasibility of RNAseq in PBMCs, a new protocol which is more sensitive than microarray and less invasive than biopsy, to understand the genetic signatures of kidney transplantation.

## Supporting Information

S1 Fig Expression of representative genes over time in kidney transplant recipients. Representative time series of fold expression changes relative to baseline (time 0) of genes. Each line on each graph represents the expression of the particular gene in a separate kidney allograft recipient. This figure only shows patients that have expression data for all 3 time points: baseline, week 1 and month 3 post-transplant. Some of the patients also have month 6 time point.
(TIF)

S1 Table Genes with higher levels at week 1 post-transplant compared to pre-transplant. Table shows gene ID, fold change expression compared to baseline, false discovery rate (FDR) and p-value.
(XLSX)

S2 Table Genes with lower levels at week 1 post-transplant compared to pre-transplant. Table shows gene ID, fold change expression compared to baseline, false discovery rate (FDR) and p-value.
(XLSX)

S3 Table Genes with higher levels at 3 months post-transplant compared to pre-transplant. Table shows gene ID, fold change expression compared to baseline, false discovery rate (FDR) and p-value.
(XLSX)

S4 Table Genes with lower levels at 3 months post-transplant compared to pre-transplant. Table shows gene ID, fold change expression compared to baseline, false discovery rate (FDR) and p-value.
(XLSX)

S5 Table Genes with higher levels at 6 months post-transplant compared to pre-transplant. Table shows gene ID, fold change expression compared to baseline, false discovery rate (FDR) and p-value.
(XLSX)

S6 Table Genes with lower levels at 6 months post-transplant compared to pre-transplant. Table shows gene ID, fold change expression compared to baseline, false discovery rate (FDR) and p-value.
(XLSX)

S7 Table Differentially expressed genes in pathways. (a) At week 1, (b) at 3 months, (c) at 6 months.
(XLSX)

S8 Table Ingenuity Pathway Analysis of Pathways. Molecular Cellular Pathways associated with kidney transplantation when analyzing the genes with higher levels compared to baseline, genes with lower levels or genes with higher and lower levels, combined.
(DOCX)
