# Supplementary material for: Differentially Expressed Gene Transcripts Using RNA Sequencing from the Blood of Immunosuppressed Kidney Allograft Recipients
Source: PLoS One. 2015 May 6;10(5):e0125045. doi: 10.1371/journal.pone.0125045 (PMC4422721; doi:10.1371/journal.pone.0125045)
Supplement: S8 Table — Molecular Cellular Pathways associated with kidney transplantation when analyzing the genes with higher levels compared to baseline, genes with lower levels or genes with higher and lower levels, combined. (DOCX) [file pone.0125045.s009.docx]

| **Week 1** | **Month 3** | **Month 6** |
| --- | --- | --- |
| **Higher Levels Than at Baseline** | | |
| Axonal Guidance Signaling | IL-8 Signaling | Antiproliferative Role of Somatostatin Receptor 2 |
| LXR/RXR Activation | Complement System | Spermine and Spermidine Degradation I |
| Airway Pathology in Chronic Obstructive Pulmonary Disease | Airway Pathology in Chronic Obstructive Pulmonary Disease | Melatonin Degradation II |
| Complement System | P2Y Purigenic Receptor Signaling Pathway | Breast Cancer Regulation by Stathmin1 |
| Inhibition of Matrix Metalloproteases | Extrinsic Prothrombin Activation Pathway | Tyrosine Degradation I |
| Neuroprotective role of THOP1 in Alzheimer’s disease | Axonal Guidance Signaling | Axonal Guidance Signaling |
| IL-8 signaling | Melatonin Degradation III | Bladder Cancer Signaling |
| Extrinsic Prothrombin Activation Pathway | Granulocyte Adhesion and Diapedesis | Glioma Signaling |
| Melotonin Degradation | Bladder Cancer Signaling | Pancreatic Adenocarcinoma Signaling |
| Superpathway of Melotonin Degradation | Agranulocyte Adhesion and Diapedesis | Role of IL-17A in Psoriasis |
| **Lower Levels Than at Baseline** | | |
| T Cell Receptor Signaling | T Cell Receptor Signaling | Granulocyte Adhesion and Diapedesis |
| iCOS-iCOSL Signaling in T Helper Cells | iCOS-iCOSL Signaling in T Helper Cells | Agranulocyte Adhesion and Diapedesis |
| CTLA4 Signaling in Cytotoxic T lymphocytes | CTLA4 Signaling in Cytotoxic T Lymphocytes | Communication between Innate and Adaptive Immune Cells |
| CD28 Signaling in T-helper Cells | CD28 Signaling in T Helper Cells | Role of Hypercytokinemia/hyperchemokinemia in the Pathogenesis of Influenza |
| Natural Killer Cell Signaling | Calcium-induced T Lymphocyte Apoptosis | Pathogenesis of Multiple Sclerosis |
| Cytotoxic Tlymphocyte-mediated Apoptosis of Target Cells | Primary Immunodeficiency Signaling | CCR5 Signaling in Macrophages |
| Calcium Induced Tlymphocytes Apoptosis | Role of NFAT in Regulation of the Immune Response | TREM1 Signaling |
| Regulation of IL-2 Expression in Activated and Anergic T Lymphocytes | Natural Killer Cell Signaling | Differential Regulation of Cytokine Production in Macrophages and T Helper Cells by IL-17A and IL-17F |
| T Helper Cell Differentiation | CCR5 Signaling in Macrophages | Differential Regulation of Cytokine Production in Intestinal Epithelial Cells by IL-17A and IL-17F |
| Type I Diabetes Melitus Signaling | Cytotoxic T Lymphocyte-mediated Apoptosis of Target Cells | Hepatic Fibrosis / Hepatic Stellate Cell Activation |
| **Higher and Lower Level, Combined** | | |
| T Cell Receptor Signaling | T Cell Receptor Signaling | Granulocyte Adhesion and Diapedesis |
| iCOS-iCOSL Signaling in T Helper Cells | iCOS-iCOSL Signaling in T Helper Cells | Agranulocyte Adhesion and Diapedesis |
| CTLA4 Signaling in Cytotoxic T-lymphocytes | CTLA4 Signaling in Cytotoxic T Lymphocytes | Communication between Innate and Adaptive Immune Cells |
| Cytotoxic T-lymphocyte-mediated Apoptosis of Target Cells | CD28 Signaling in T Helper Cells | CCR5 Signaling in Macrophages |
| CD28 Signaling in T-helper Cells | Calcium-induced T Lymphocyte Apoptosis | Pathogenesis of Multiple Sclerosis |
| Calcium Induced T-lymphocytes Apoptosis | Primary Immunodeficiency Signaling | Role of Hypercytokinemia/hyperchemokinemia in the Pathogenesis of Influenza |
| Natural Killer Cell Signaling | CCR5 Signaling in Macrophages | Role of IL-17F in Allergic Inflammatory Airway Diseases |
| Regulation of IL-2 Expression in Activated and Anergic T Lymphocytes | Role of NFAT in Regulation of the Immune Response | Role of IL-17A in Psoriasis |
| CCR5 Signaling in Macrophages | Cytotoxic T Lymphocyte-mediated Apoptosis of Target Cells | Differential Regulation of Cytokine Production in Macrophages and T Helper Cells by IL-17A and IL-17F |
| T Helper Cell Differentiation | Natural Killer Cell Signaling | Hepatic Fibrosis / Hepatic Stellate Cell Activation |

**Supplemental Figure 8** Ingenuity Pathway Analysis of Pathways when analyzing the top genes with higher levels than baseline, lower levels and combined higher and lower levels.
